# MicroRNA-130a alleviates human coronary artery endothelial cell injury and inflammatory responses by targeting PTEN via activating PI3K/Akt/eNOS signaling pathway

**DOI:** 10.18632/oncotarget.12431

**Published:** 2016-10-04

**Authors:** Chun-Li Song, Bin Liu, Yong-Feng Shi, Ning Liu, You-You Yan, Ji-Chang Zhang, Xin Xue, Jin-Peng Wang, Zhuo Zhao, Jian-Gen Liu, Yang-Xue Li, Xiao-Hao Zhang, Jun-Duo Wu

**Affiliations:** ^1^ Department of Cardiology, The Second Hospital of Jilin University, Changchun 130041, Jilin Province, China

**Keywords:** microRNA-130a, PTEN, PI3K/Akt/eNOS signaling pathway, coronary artery endothelial cells, cell injury

## Abstract

Our study aims to investigate the roles of microRNA-130a (miR-130a) in human coronary artery endothelial cells (HCAECs) injury and inflammatory responses by targeting PTEN through the PI3K/Akt/eNOS signaling pathway. HCAECs were treated with 1.0 mmol/L homocysteine (HCY) and assigned into eight groups: the blank group, the negative control (NC) group, the miR-130a mimics group, the miR-130a inhibitors group, the si-PTEN group, the Wortmannin group, the miR-130a inhibitors + si-PTEN group and the miR-130a mimics + Wortmannin group. Luciferase reporter gene assay was used to validate the relationship between miR-130a and PTEN. The expressions of miR-130a, PTEN and PI3K/Akt/eNOS signaling pathway-related proteins were detected by qRT-PCR assay and Western blotting. MTT assay and Hoechst 33258 staining were adopted to testify cell growth and apoptosis. The NO kit assay was used to detect the NO release. ELISA was conducted to measure serum cytokine levels. Luciferase reporter gene assay confirmed the target relationship between miR-130a and PTEN. Compared with the blank and NC groups, the miR-130a mimics and si-PTEN groups showed significant increases in the expressions of PI3K/Akt/eNOS signaling pathway-related proteins, cell viability and the NO release, while serum cytokine levels and cell apoptosis were decreased; by contrast, an opposite trend was observed in miR-130a inhibitors and Wortmannin groups. However, no significant difference was found in the miR-130a inhibitors + si-PTEN and miR-130a mimics + Wortmannin groups when compared with the blank group. These results indicate that miR-130a could alleviate HCAECs injury and inflammatory responses by down-regulating *PTEN* and activating PI3K/Akt/eNOS signaling pathway.

## INTRODUCTION

Endothelial cells (ECs) have a vigorous ability to grow and are involved in angiogenesis, which comprises both neovascularization and the maintenance of intimal layer integrity [1]. The proliferation of ECs plays an important role in angiogenesis, which is a physiological or pathological neovascularization process that occurs under certain conditions, such as tissue ischemia [2]. ECs have also play a critical role in the vascular and inflammatory responses, and the activation of inflammatory in ECs is the key to the development of many vascular diseases, such as vascular permeability and endothelial hyper-permeability [3, 4]. ECs can secrete a variety of vasoactive substances, such as ET and nitric oxide (NO), which can participate in various signaling pathways, thereby regulating endothelial function [5, 6]. It has been demonstrated that activation of the PI3K/Akt pathway and the eNOS/NO pathway are closely associated with vascular remodeling and angiogenesis [7]. Previous evidence has reported that the activation of the PI3K/Akt/eNOS signaling pathway can promote NO release and thus inhibit pro-inflammatory cytokines production [8, 9].

MicroRNA-130a (miR-130a) is located at chromosome 11q12, close to the 11q13 area [10]. It has been proposed that miR-130a can promote the proliferation, migration and tube formation of vascular endothelial cells [11]. MiR-130a has recently been found to be involved in many critical processes in multiple types of human diseases, including ventricular arrhythmias, endothelial progenitor cell dysfunction and hepatic insulin sensitivity [12–15]. Homocysteine (HCY) is capable of inducing the apoptosis in endothelial cells, and it could be an essential mechanism for development of cardiovascular diseases [16]. Previous studies have demonstrated that miRs are involved in several HCY-induced cardiovascular dysfunctions, including cardiac remodeling and stroke [17, 18]. The physiological function of phosphatase and tensin homologue (PTEN), a lipid phosphatase, is a frequently mutated tumor suppressor gene that opposes the PI3K/AKT pathway through dephosphorylation of phosphoinositide-3,4,5-triphosphate [19, 20]. In the present study, we aim to investigate the roles of miR-130a in HCAECs injury and inflammatory responses by targeting PTEN through the PI3K/Akt/eNOS signaling pathway.

## RESULTS

### The relationship between miR-130a and PTEN

**Figure 1:**
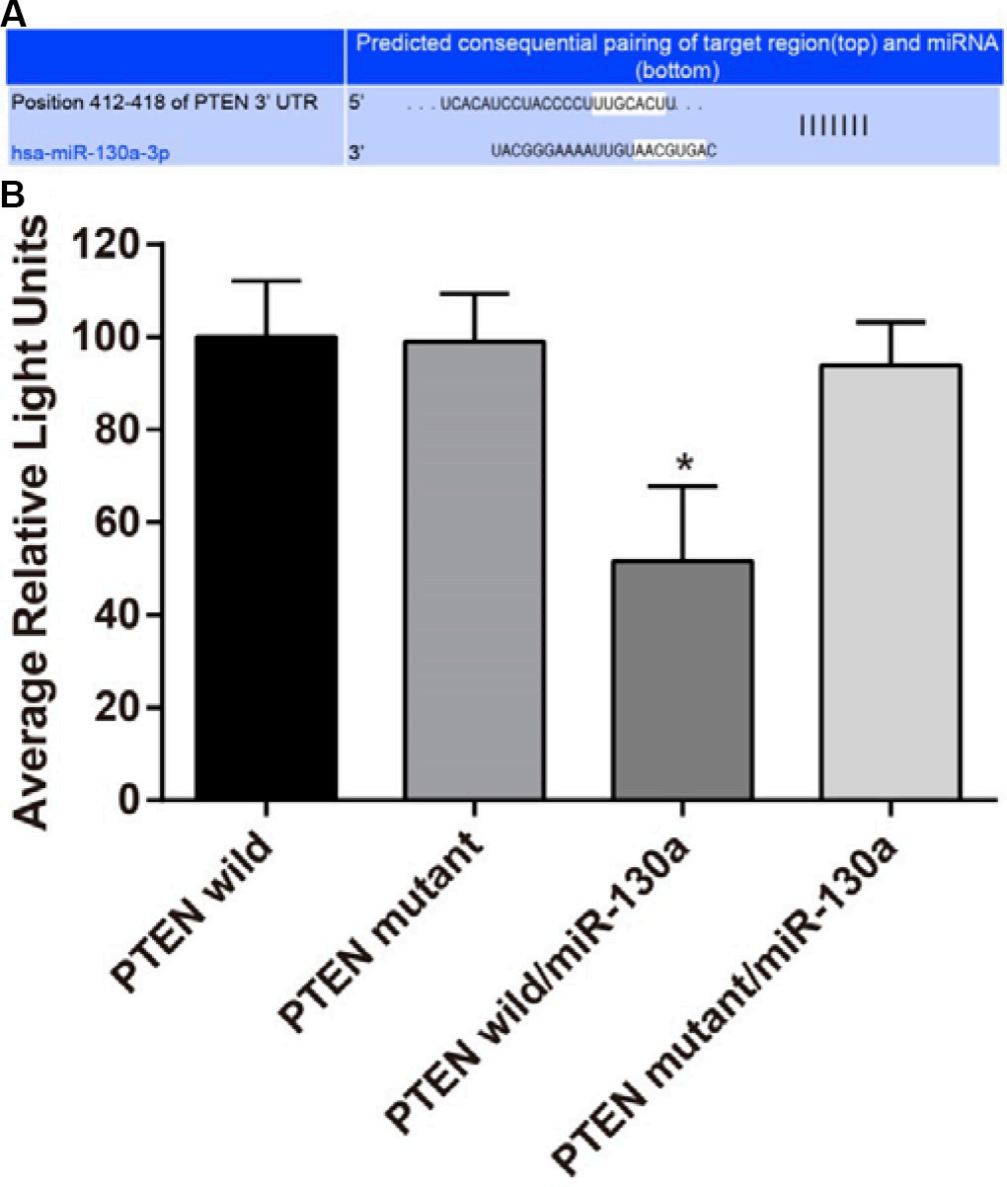
MiR-130a directly target at *PTEN* (**A**) Target Scan used for predicting the 3′-UTR primers of miR-130a binding site with PTEN mRNA; (**B**) Dual luciferase reporter gene activity assay. Note: PTEN, phosphatase and tensin homolog deleted on chromosome 10; *P < 0.05 compared with the PTEN wild.

The target site related to PTEN and miR-130a was determined via Target Scan software (http://www.targetscan.org/vert_61/). Figure 1 showed the 3′-UTR primers of miR-130a binding site with PTEN mRNA. In order to prove that the change of luciferase activity was caused by binding site predicted by the miR-130a, we also designed the mutant and wild sequences which were loss the miR-130a binding site in PTEN 3′UTR, and inserted into reporter plasmid. The 293T cell was co-transfected with different plasmids (PTEN wild group, PTEN mutant group, PTEN wild/miR-130a group and PTEN mutant/miR-130a group). Luciferase activity was conducted for testing, and the result showed that compared with the other transfection groups, PTEN wild/miR-130a group showed lower luciferase activity (all *P* < 0.05). MiR-130a can specifically inhibit the expression of UTR 3′ region of PTEN.

### Effects of different doses of HCY on cell viability of HCAECs

**Figure 2:**
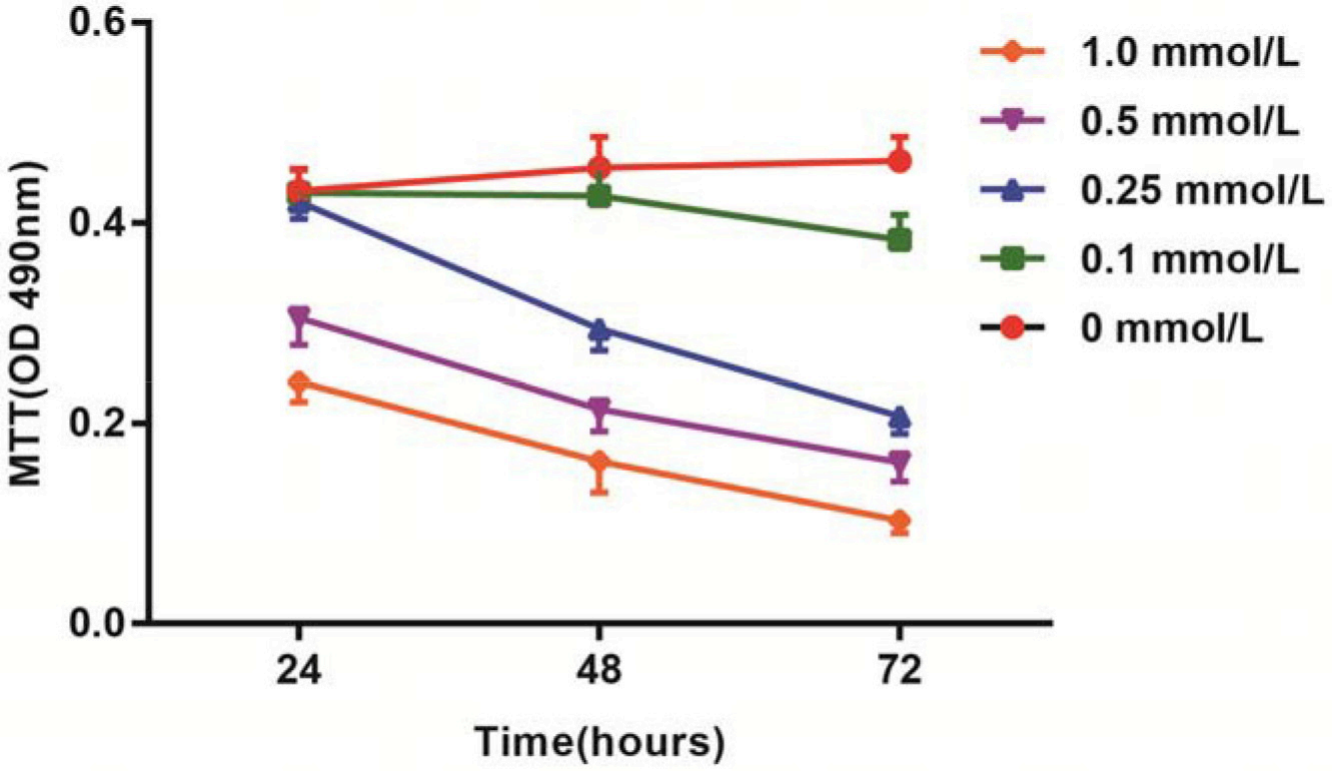
Effects of different doses (0, 0.1, 0.25, 0.5 and l.0 mmol/L) of HCY on cell activity of HCAECs Note: HCY, homocysteine; HCAECs, human coronary artery endothelial cells.

After treatment with different doses of HCY for 24 h, 48 h and 72 h, the activation of HCAECs was detected using an MTT assay. The results showed that the activation of HCAECs receiving HCY concentrations of 0.1, 0.25, 0.5 or l.0 mmol/L was decreased, and in 24 h, a significant difference was found between the proliferation rate of HCAECs treated with HCY concentrations of 0.5 and l.0 mmol/L and that of the control group (HCY concentration 0 mmol/L) (both *P* < 0.01). Compared with the control group (0 mmol/L), the cell proliferation rates after HCY treatment (0.25, 0.5 and l.0 mmol/L) were significantly different at 48 h (all *P* < 0.05). Compared with the control group (0 mmol/L), the cell proliferation rates after HCY treatment (0.1, 0.25, 0.5 and l.0 mmol/L) were significantly different at 48 h (all *P* < 0.05) (Figure 2).

### Effects of different doses of HCY on cell apoptosis of HCAECs

**Figure 3:**
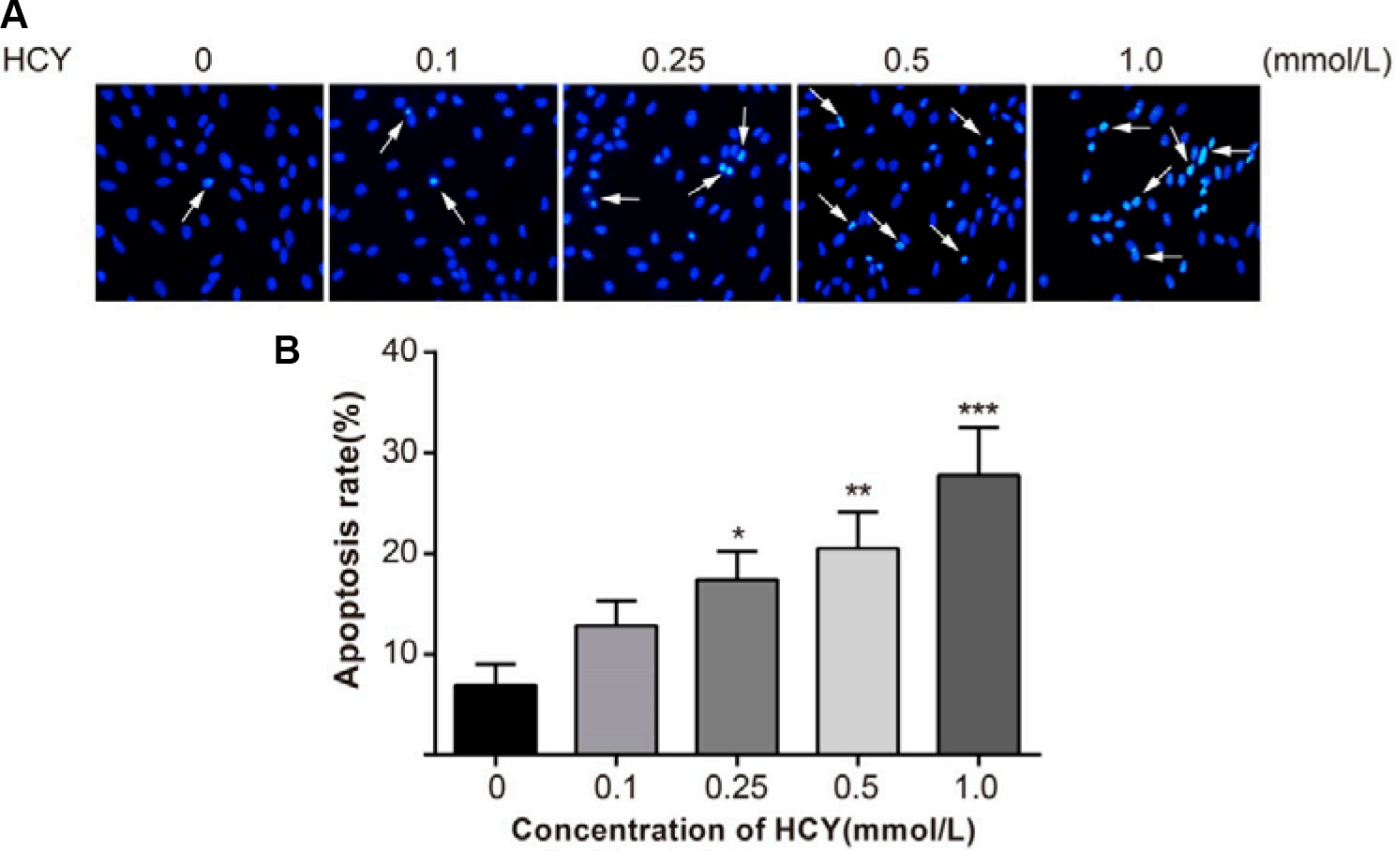
Effect of different doses (0, 0.1, 0.25, 0.5 and l.0 mmol/L) of HCY on cell apoptosis of HCAECs (**A**) A series of typical apoptosis characteristics of HCAECs, such as chromatin enrichment and apoptotic body after treating by difference concentrations of HCY (×200); (**B**) The effects of different doses (0, 0.1, 0.25, 0.5 and l.0 mmol/L) of HCY on apoptosis of HCAECs, compared with the control group (0 mmol/L). Note: HCY, homocysteine; HCAECs, human coronary artery endothelial cells; *P < 0.05, **P < 0.01, ***P < 0.001.

Hoechst 33258 is a nucleic acid-specific dye. The nuclei in normal cells presented uniformly hypochromatic blue color, and the nuclei in apoptotic cells presented a series of typical apoptosis characteristics, such as pyknosis, chromatin enrichment and apoptotic bodies (Figure 3A). After treatment with different doses of HCY (0.1, 0.25, 0.5 and l.0 mmol/L) for 24 h, the apoptosis rates (12.8 ± 2.5%, 17.4 ± 2.8%, 20.5 ± 3.6%, 27.8 ± 4.7%, respectively) were significantly increased compared with the apoptosis rate (6.9 ± 2.1%) in the control group (0 mmol/L) (all *P* < 0.05) (Figure 3B).

### Effects of different doses of HCY on the release of NO in HCAECs

**Figure 4:**
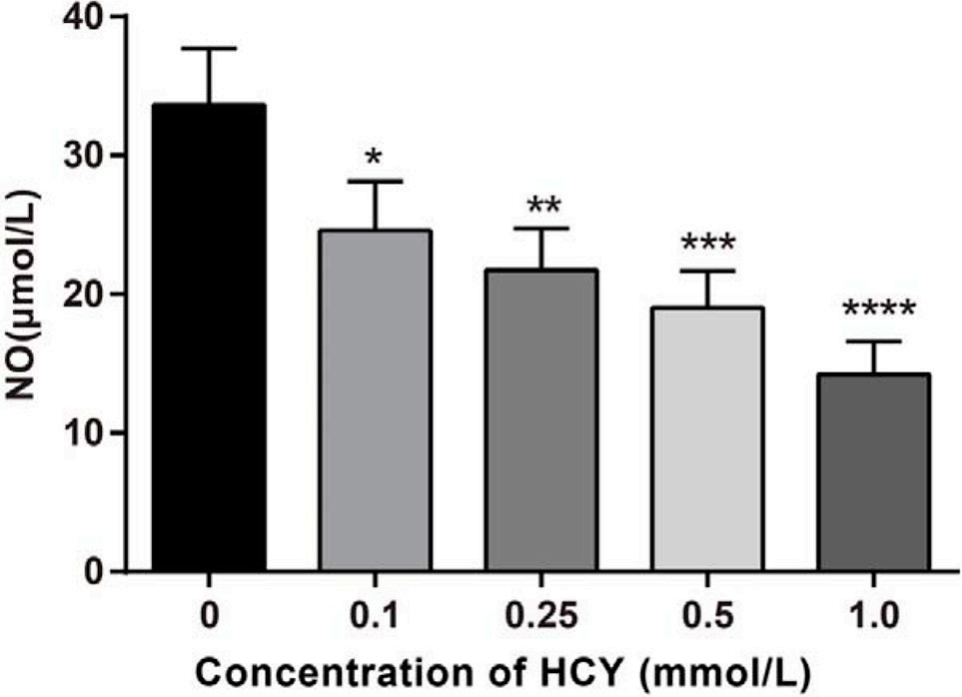
Effect of different doses (0, 0.1, 0.25, 0.5 and l.0 mmol/L) of HCY on the release of NO of HCAECs Note: HCY, homocysteine; HCAECs, human coronary artery endothelial cells; NO, Nitric oxide; compared with the control group (0 mmol/L), *P < 0.05, **P < 0.01, ***P < 0.001, ****P < 0.0001.

A NO kit was used to detect the NO content in the cell supernatant. The results showed that the concentrations of NO were 24.58 ± 3.52 mmol/L, 21.74 ± 3.02 mmol/L, 19.02 ± 2.65 mmol/L, 14.23 ± 2.36 mmol/L, respectively, after treatment with different doses of HCY (0.1, 0.25, 0.5 and l.0 mmol/L), which were lower than the concentration of NO (33.65 ± 4.08 mmol/L) in the control group (0 mmol/L) (all *P* < 0.05) (Figure 4).

### Effects of different doses of HCY on serum cytokine levels in HCAECs

The ICAM-1 and IL-6 content in HCAECs treated with different doses of HCY (0.1, 0.25, 0.5 and l.0 mmol/L) was detected by ELISA. The results showed that with increasing HCY concentrations, the ICAM-1, IL-6, IFN-γ, IL-1β, TNF-α, IL-12 and IL-17 contents in HCAECs increased gradually. The IL-6, ICAM-1, IFN-γ, IL-1β, TNF-α, IL-12 and IL-17 contents in HCAECs treated with HCY at concentrations of 0.25, 0.5 and l.0 mmol/L were significantly different than those of the control group (0 mmol/L), except for the 0.1 mmol/L HCY condition (all *P* < 0.05) (Table 1).

**Table 1:** Effect of different concentrations of HCY on inflammatory cytokines secreted by HCAECs

HCY concentration (mmol/L)	IL-6 (pg/ml)	ICAM-1 (ng/ml)	IFN-γ (pg/ml)	IL-1β′ (pg/ml)	TNF-α (pg/ml)	IL-12 (pg/ml)	IL-17 (pg/ml)
0	10.25 ± 4.18	4.24 ± 1.34	28.86 ± 3.64	1.36 ± 0.47	1.85 ± 0.79	11.43 ± 4.67	3.93 ± 1.45
0.1	12.64 ± 4.72	5.37 ± 2.45	30.74 ± 4.39	1.67 ± 0.56	2.04 ± 0.90	14.90 ± 4.82	4.25 ± 1.33
0.25	30.14 ± 5.02*	13.24 ± 3.08*	42.63 ± 3.9^5^*	2.79 ± 1.23*	5.26 ± 1.27*	37.47 ± 7.65*	6.68 ± 2.02*
0.5	41.32 ± 5.89*	16.21 ± 3.21*	55.48 ± 4.67*	4.31 ± 1.28*	7.14 ± 2.16*	51.38 ± 8.44*	8.59 ± 2.61*
1	57.88 ± 7.66*	21.33 ± 3.64*	70.15 ± 5.01*	6.47 ± 1.59*	11.56 ± 3.73*	71.24 ± 10.52*	12.76 ± 4.18*

Notes: HCY, homocysteine; IL-6, interleukin-6; ICAM-1, Intercellular Adhesion Molecule 1; IFN-γ, interferon-γ; IL-1β, Interleukin-1β; TNF-α, interferon-α; IL-12, interleukin-12; IL-17, interleukin-17; HCAECs: human coronary artery endothelial cells;

* compared with the control group (0 mmol/L), *P* < 0.05.

### Effects of different doses of HCY on miR-130a and PTEN mRNA expressions in HCAECs

**Figure 5:**
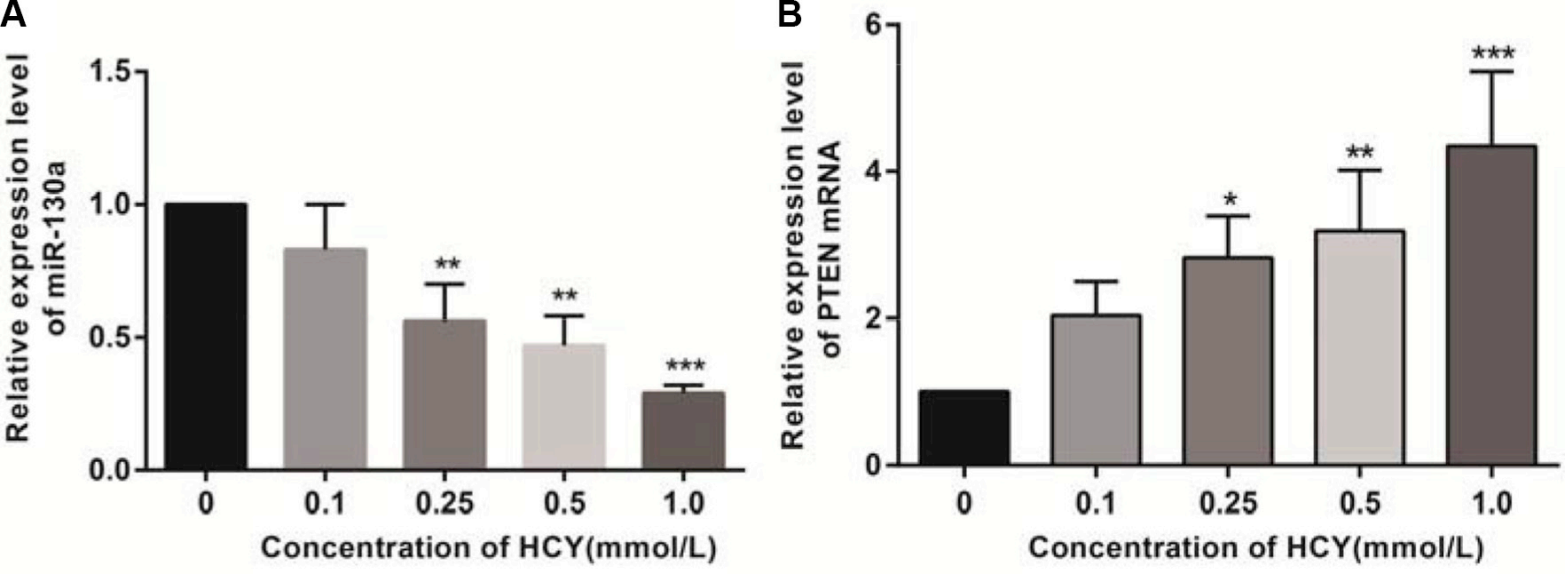
Effect of different doses (0, 0.1, 0.25, 0.5 and l.0 mmol/L) of HCY on the expressions of miR-130a and PTEN mRNA in HCAEC cells detected by RT-qPCR (**A**) Effect of different doses (0, 0.1, 0.25, 0.5 and l.0 mmol/L) of HCY on the relative expression of miR-130a in HCAEC cells; (**B**) Effect of different doses (0, 0.1, 0.25, 0.5 and l.0 mmol/L) of HCY on the relative expression of PTEN mRNA in HCAEC cells. Note: HCY, homocysteine; HCAECs, human coronary artery endothelial cells; PTEN, phosphatase and tensin homolog deleted on chromosome 10; compared with the control group (0 mmol/L), **P < 0.01, ***P < 0.001.

Compared with the control group (0 mmol/L), the relative expression of miR-130a in the cell supernatant of HCY-treated cells was decreased while the expression of PTEN mRNA was increased. The relative expressions of miR-130a in the supernatants of cells treated with HCY at concentrations of 0.25, 0.5 and l.0 mmol/L were significantly lower while the expression of PTEN mRNA was higher than those in the control group (0 mmol/L), except for the 0.1 mmol/L HCY condition (*P* < 0.05), and the content of miR-130a in the cell supernatant decreased with increasing HCY concentrations while the expression of PTEN mRNA increased with increasing HCY concentrations (both *P* < 0.05) (Figure 5).

### Effects of different doses of HCY on the expressions of PTEN and PI3K-Akt-eNOS signaling pathway-related proteins in HCAECs

**Figure 6:**
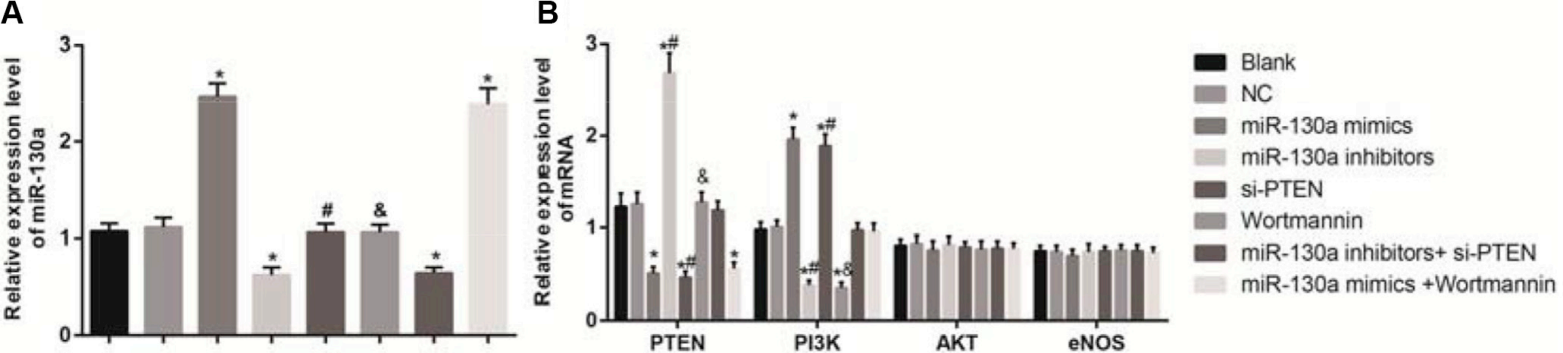
Effects of different doses (0, 0.1, 0.25, 0.5 and l.0 mmol/L) of HCY on the mRNA expressions of miR-130 and key molecules of PI3K-Akt-eNOS signaling pathway in HCAECs detected by qRT-PCR Note: (**A**) the mRNA expressions of miR-130 in HCAECs detected by qRT-PCR; B, the mRNA expressions of PTEN and the key molecules of PI3K-Akt-eNOS signaling pathway in HCAECs detected by qRT-PCR; HCAECs, Human coronary artery endothelial cells; HCY, homocysteine; PTEN, phosphatase and tensin homolog deleted on chromosome 10; PI3K, phosphatidylinositol 3-kinase; Akt: protein kinase (**B**) eNOS: endothelial nitric oxide synthase; compared with the blank and the NC groups, *P < 0.05; compared with the miR-130a inhibitors + si-PTEN group, ^#^*P* < 0.05; compared with the miR-130a mimics + Wortmannin group, ^&^*P* < 0.05).

As shown in Figure 6, the mRNA expressions of miR-130a, PTEN, PI3K, AKT and eNOS in HCAECs of each group were detected by qRT-PCR. The results demonstrated that compared with the blank and the NC groups, the mRNA expressions of miR-130a in the miR-130a mimics and the miR-130a mimics +Wortmannin groups were significantly increased while were significantly decreased in the miR-130a inhibitors and the miR-130a inhibitors + si-PTEN groups (all *P* < 0.05). Compared with the blank and the NC groups, the mRNA expressions of PTEN in the miR-130a mimics, the si-PTEN and the miR-130a mimics +Wortmannin groups were significantly increased while were significantly decreased in the miR-130a inhibitors group (all *P* < 0.05). No significant difference was found in the mRNA expression of PTEN between the Wortmannin and the miR-130a inhibitors + si-PTEN groups (*P* > 0.05). The mRNA expressions of PI3K were significantly higher in the miR-130a mimics and the si-PTEN groups while were significantly lower in the miR-130a inhibitors and the Wortmannin groups when compared with those in the blank and the NC groups (all *P* < 0.05). Compared with the miR-130a inhibitors + si-PTEN group, the mRNA expression of PI3K was lower in the miR-130a inhibitors group while were higher in the si-PTEN group (both *P* < 0.05). Compared with the miR-130a mimics +Wortmannin group, the mRNA expression of PI3K was higher in the miR-130a mimics group while were lower in the Wortmannin group (both *P* < 0.05). However, no significant difference was found in the mRNA expressions of AKT and eNOS among cells in each group (all *P* > 0.05).

**Figure 7:**
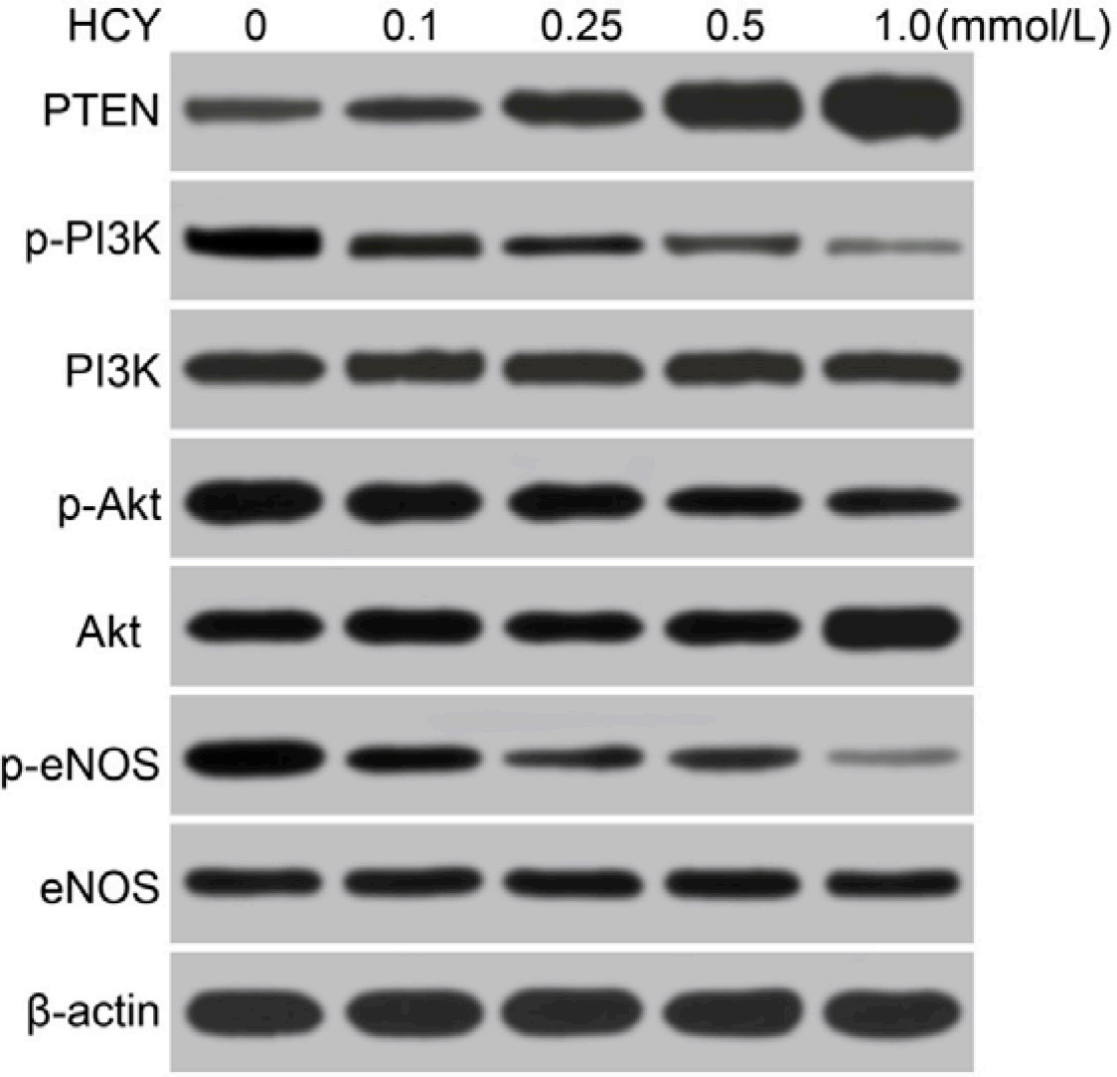
Effects of different doses (0, 0.1, 0.25, 0.5 and l.0 mmol/L) of HCY on the expressions of PTEN and the PI3K/Akt/eNOS signaling pathway-related proteins in HCAECs detected by Western blotting Note: HCAECs, Human coronary artery endothelial cells; HCY, homocysteine; PTEN, phosphatase and tensin homolog deleted on chromosome 10; PI3K, phosphatidylinositol 3-kinase; Akt: protein kinase B; eNOS: endothelial nitric oxide synthase.

Western blotting analysis revealed that the p-PI3K, p-Akt, p-eNOS, p-PI3K/PI3K, p-Akt/Akt and p-eNOS/eNOS were gradually decreased, and the PTEN was gradually increased in HCAECs treated with different doses of HCY (0.1, 0.25, 0.5 and l.0 mmol/L), and a significant difference was found compared with the control group (0 mmol/L) (all *P* < 0.05). Based on the above results, the high level of HCY was an independent risk factor for coronary heart disease (CHD), and miR-130a expression was decreased, and PTEN expression was increased in HCAECs treated with high concentrations of HCY, which then inhibited the PI3K-Akt-eNOS signaling pathway. Thus, for the following experiments treatment of HCAECs with 1.0 mmol/L HCY or PBS was used to further explore whether miR-130a promoted the function of the PI3K/Akt/eNOS signaling pathway in HCAEC injury and HCAEC-mediated inflammation via targeting PTEN (Figure 7 and Table 2).

**Table 2:** Effects of different concentrations (0, 0.1, 0.25, 0.5 and l.0 mmol/L) of HCY on expressions of PETN, key molecules of PI3K-Akt-eNOS signaling pathway and its phosphorylated proteins in HCAECs

HCY concentration (mmol/L)	PTEN	p-PI3K	PI3K	p-Akt	Akt	p-eNOS	eNOS	p-PI3K/PI3K	p-Akt/Akt	p-eNOS/eNOS
0	5.26 ± 1.83	3.15 ± 0.29	4.02 ± 0.54	2.66 ± 0.21	3.34 ± 0.20	2.78 ± 0.35	3.41 ± 0.31	0.786 ± 0.034	0.796 ± 0.013	0.813± 0.028
0.1	6.08 ± 1.95	2.46 ± 0.25*	3.91 ± 0.41	1.88 ± 0.18*	3.56 ± 0.36	1.97 ± 0.28*	3.24 ± 0.28	0.631 ± 0.074*	0.528 ± 0.002*	0.606 ± 0.033*
0.25	7.27± 1.88*	1.80 ± 0.22*	3.88 ± 0.43	1.68 ± 0.27*	3.48 ± 0.38	1.52 ± 0.24*	3.19 ± 0.24	0.465 ± 0.048*	0.474 ± 0.053*	0.478 ± 0.087*
0.5	7.93± 2.14*	1.35 ± 0.23*	3.89 ± 0.36	1.26 ± 0.25*	3.53 ± 0.18	1.12 ± 0.21*	3.21 ± 0.33	0.344 ± 0.028*	0.355 ± 0.054*	0.347 ± 0.029*
1	9.13 ± 2.30*	1.01 ± 0.18*	3.85 ± 0.37	0.81 ± 0.24*	3.31 ± 0.27	0.68 ± 0.18*	3.15 ± 0.41	0.262 ± 0.022*	0.243 ± 0.075*	0.214 ± 0.026*

Notes: HCY: Homocysteine; HCAECs: Coronary artery endothelial cells; PTEN; Phosphatase and tensin homolog; PI3K, Phosphatidylinositol 3-kinase; Akt, Protein kinase B; eNOS, endothelial nitric oxide synthase

* compared with the control group (0 mmol/L), *P* < 0.05.

### Comparisons of the expressions of miR-130a, PTEN and the PI3K/Akt/eNOS signaling pathway-related proteins in HCAECs among eight groups

**Figure 8:**
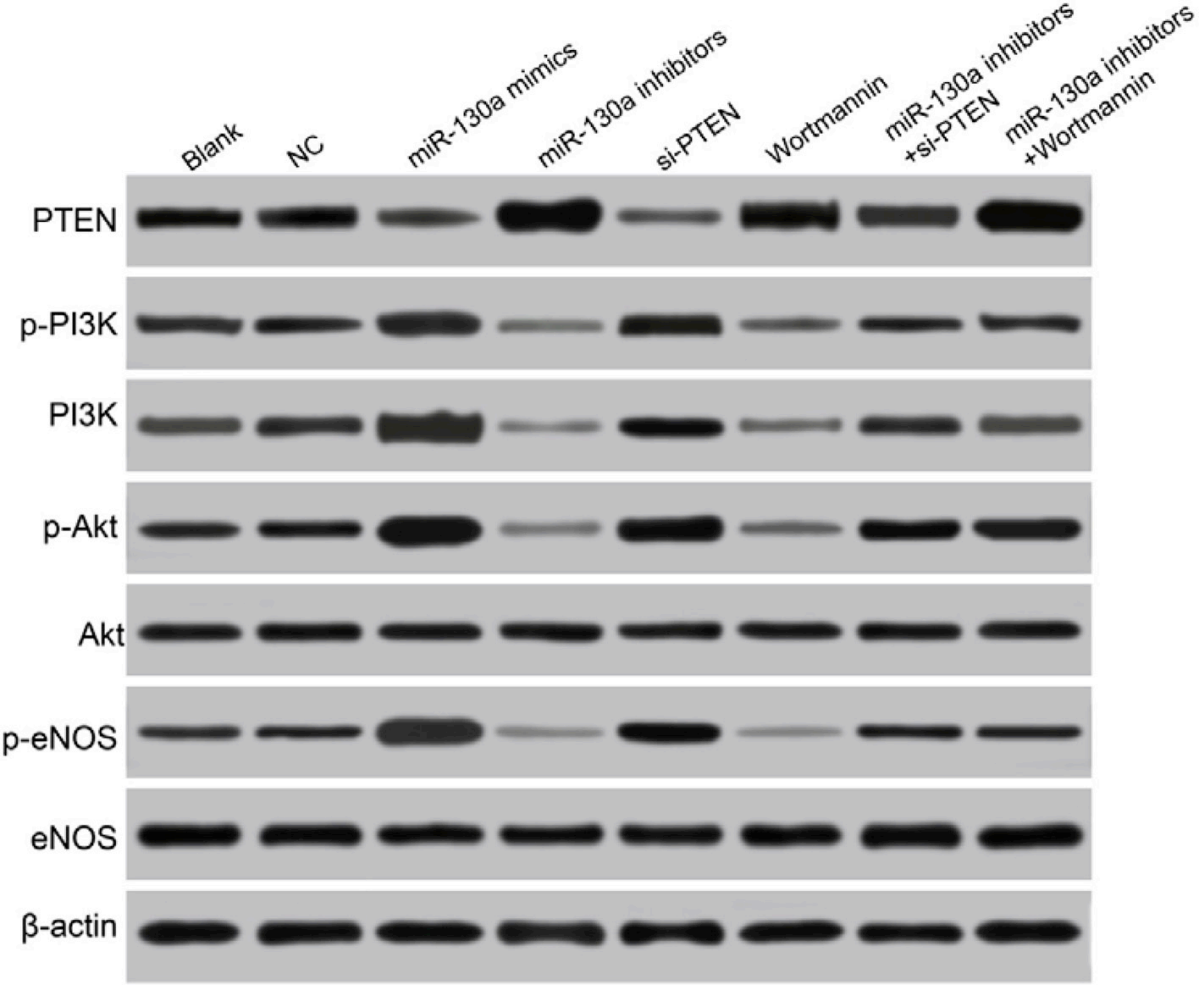
Comparisons of the expressions of miR-130a, PTEN and the PI3K/Akt/eNOS signaling pathway-related proteins in HCAECs among eight groups Note: HCAECs, human coronary artery endothelial cells; PTEN, phosphatase and tensin homolog; PI3K, phosphatidylinositol 3-kinase; Akt, protein kinase B; eNOS, endothelial nitric oxide synthase.

Western blotting analysis results demonstrated that compared with the blank and the NC groups, the protein expressions of PTEN were significantly increased in the miR-130a mimics, si-PTEN and miR-130a mimics +Wortmannin groups, and the protein expression of PTEN was significantly decreased in the miR-130a inhibitors group (all *P* < 0.05), while no significant difference was found between the Wortmannin group and the miR-130a inhibitors + si-PTEN group (*P* > 0.05). Compared with the blank and the NC groups, the protein expressions of PI3K, p-PI3K, p-Akt, and p-eNOS and the ratios of p-PI3K/PI3K, p-Akt/Akt and p-eNOS/eNOS were higher in the miR-130a mimics and the si-PTEN group while were lower in the miR-130a inhibitors and the Wortmannin group (all *P* < 0.05). Compared with the miR-130a inhibitors + si- PTEN group, the protein expressions of PI3K, p-PI3K, p-Akt, and p-eNOS and the ratios of p-PI3K/PI3K, p-Akt/Akt and p-eNOS/eNOS were significantly decreased in the miR-130a inhibitors group while increased in the si-PTEN group (all *P* < 0.05). Compared with the miR-130a mimics +Wortmannin group, the protein expressions of PI3K, p-PI3K, p-Akt, and p-eNOS and the ratios of p-PI3K/PI3K, p-Akt/Akt and p-eNOS/eNOS were significantly increased in the miR-130a mimics group while decreased in the Wortmannin group (all *P* < 0.05). Compared with the blank and the NC groups, no significant difference was found for the protein expressions of PI3K, p-PI3K, p-Akt, and p-eNOS and the ratios of p-PI3K/PI3K, p-Akt/Akt and p-eNOS/eNOS in the miR-130a inhibitors + si-PTEN and the miR-130a mimics +Wortmannin groups (Figure 8 and Table 3).

**Table 3:** Expressions of PETN, key molecules of PI3K-Akt-eNOS signaling pathway and its phosphorylated proteins in HCAECs of each group

Group	PTEN	p-PI3K	PI3K	p-Akt	Akt	p-eNOS	eNOS	p-PI3K/PI3K	p-Akt/Akt	p-eNOS/eNOS
Blank	1.59 ± 0.47	0.73 ± 0.12	1.63 ± 0.14	0.56 ± 0.08	1.14 ± 0.11	0.57 ± 0.09	1.12 ± 0.11	0.447 ± 0.035	0.489 ± 0.028	0.509 ± 0.057
NC	1.52 ± 0.44	0.79 ± 0.10	1.67 ± 0.13	0.58 ± 0.10	1.18 ± 0.10	0.59 ± 0.10	1.13 ± 0.10	0.469 ± 0.025	0.490 ± 0.046	0.518 ± 0.049
miR-130a mimics	0.59 ± 0.20*	1.58 ± 0.23*^&^	2.49 ± 0.18*^&^	0.87 ± 0.10*^&^	1.17 ± 0.13	0.95 ± 0.12*^&^	1.16 ± 0.12	0.633 ± 0.048*^&^	0.744 ± 0.007*^&^	0.820 ± 0.016*^&^
miR-130a inhibitors	2.02 ± 0.39*^#^	0.32 ± 0.07*^#^	0.57 ± 0.14*^#^	0.23 ± 0.05*^#^	1.18 ±0.07	0.18 ± 0.06*^#^	1.13 ± 0.11*^#^	0.560 ± 0.018*^#^	0.194 ± 0.031*^#^	0.160 ± 0.037*^#^
si-PTEN	0.53 ± 0.17*^#^	1.62 ± 0.20*^#^	2.51 ± 0.21*^#^	0.92 ± 0.10*^#^	1.20 ± 0.11	0.97 ± 0.12*^#^	1.19 ± 0.13*^#^	0.644 ± 0.028*^#^	0.764 ± 0.011*^#^	0.817 ± 0.011*^#^
Wortmannin	1.63 ± 0.53^&^	0.27 ± 0.08*^&^	0.51 ± 0.11*^&^	0.20 ± 0.06*^&^	1.16 ±0.05	0.16 ± 0.05*^&^	1.11 ± 0.11*^&^	0.526 ± 0.052*^&^	0.174 ± 0.041*^&^	0.142 ± 0.035*^&^
miR-130a inhibitors + si-PTEN	1.47 ± 0.46	0.74 ± 0.14	1.63 ± 0.22	0.64 ± 0.09	1.16 ± 0.11	0.67 ± 0.14	1.13 ± 0.13	0.453 ± 0.026	0.547 ± 0.032	0.592 ± 0.058
miR-130a mimics + Wortmannin	0.61± 0.14*	0.71 ± 0.13	1.61 ± 0.16	0.62 ± 0.08	1.13 ± 0.10	0.66 ± 0.09	1.09 ± 0.09	0.444 ± 0.066	0.551 ± 0.047	0.602 ± 0.037

HCY: Homocysteine; HCAECs: Coronary artery endothelial cells; TEN; Phosphatase and tensin homolog; PI3K, Phosphatidylinositol 3-kinase; Akt, Protein kinase B; eNOS, endothelial nitric oxide synthase;

* compared with the blank and the NC groups, *P* < 0.05,

# compared with the miR-130a inhibitors+ si-PTEN group, *P* < 0.05;

& compared with the miR-130a mimics + Wortmannin, *P* < 0.05.

### Comparisons of the proliferation of HCAECs among eight groups

**Figure 9:**
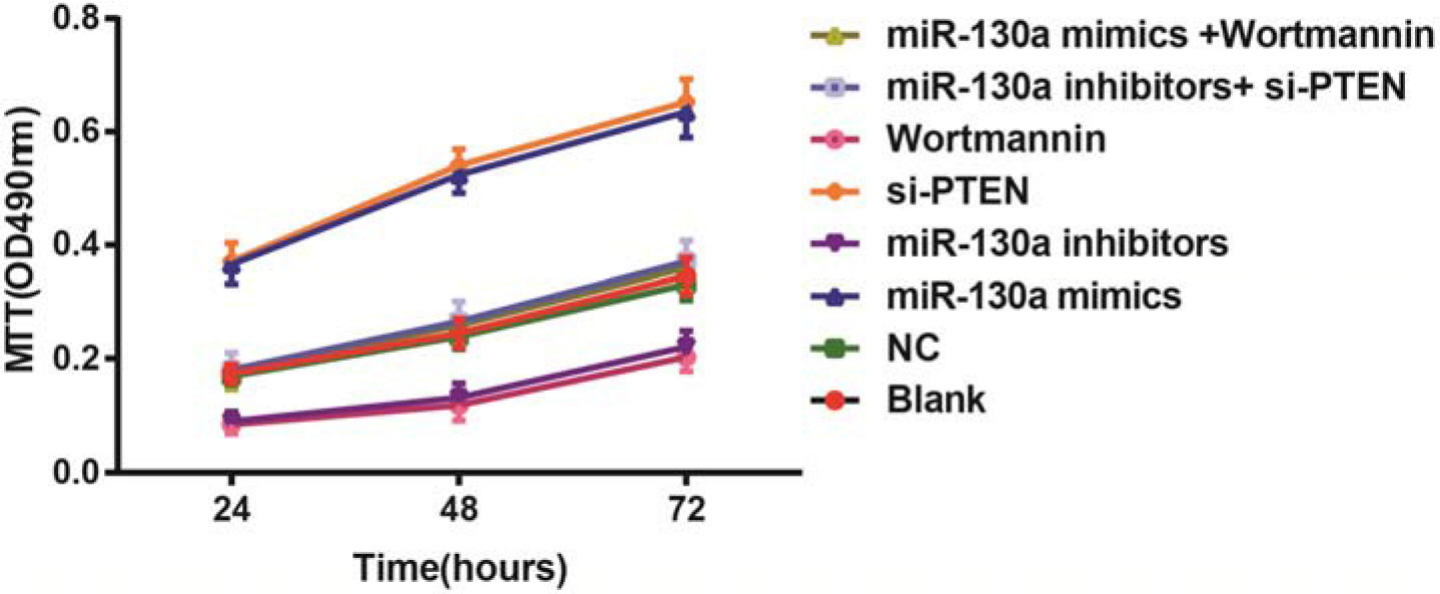
Comparisons of the proliferation of HCAECs among eight groups Note: HCAECs, Human coronary artery endothelial cells.

MTT assay revealed that compared with the blank and the NC groups, the cell activities at 24 h, 48 h and 72 h were higher in the miR-130a mimics and si-PTEN groups while were lower in the miR-130a inhibitors and the Wortmannin groups (all *P* < 0.05). The cell activity in the miR-130a mimics group was higher while in the Wortmannin group was lower than that in the miR-130a mimics +Wortmannin group (*P* < 0.05). The cell activity in the miR-130a inhibitors group was lower while in the si-PTEN group was higher than that in the miR-130a inhibitors + si-PTEN group (*P* < 0.05) (Figure 9).

### Comparisons of the apoptosis of HCAECs among eight groups

**Figure 10:**
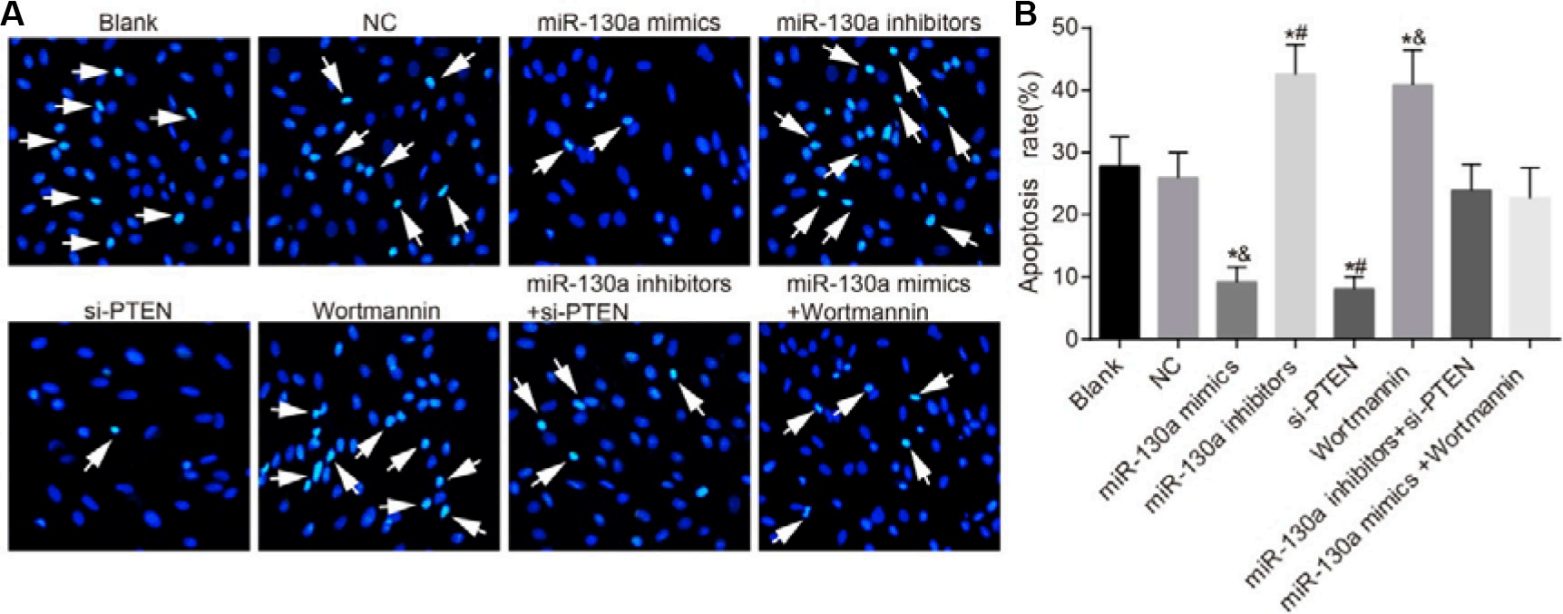
Comparisons of the apoptosis of HCAECs among eight groups (**A**) A series of typical apoptosis characteristics of HCAECs, such as chromatin enrichment and apoptotic body after treating by difference concentrations of HCY (× 200); (**B**) The effects of HCY on apoptosis of HCAECs. Note: HCAECs, human coronary artery endothelial cells; NO, nitric oxide; *P < 0.05 compared with the blank and the NC groups; ^#^*P* < 0.05 compared with the miR-130a inhibitors + si-PTEN group; ^&^*P* < 0.05 compared with the miR-130a mimics + Wortmannin group.

Hoechst 33258 staining showed that compared with the blank and the NC groups, the apoptotic rates were decreased in the miR-130a mimics and the si-PTEN groups while were increased in the miR-130a inhibitors and the Wortmannin groups (*P* < 0.05). No significant difference was found for the apoptotic rates in the miR-130a inhibitors + si-PTEN and the miR-130a mimics +Wortmannin groups in comparison to the blank and the NC groups (*P* > 0.05). The apoptotic rate was higher in the miR-130a inhibitors group while was lower in the si-PTEN group than that in the miR-130a inhibitors + si-PTEN group (*P* < 0.05). The apoptotic rate was lower in the miR-130a mimics group while was higher in the Wortmannin group than that in the miR-130a mimics +Wortmannin group (*P* < 0.05) (Figure 10).

### Comparisons of the release of NO in HCAECs among eight groups

**Figure 11:**
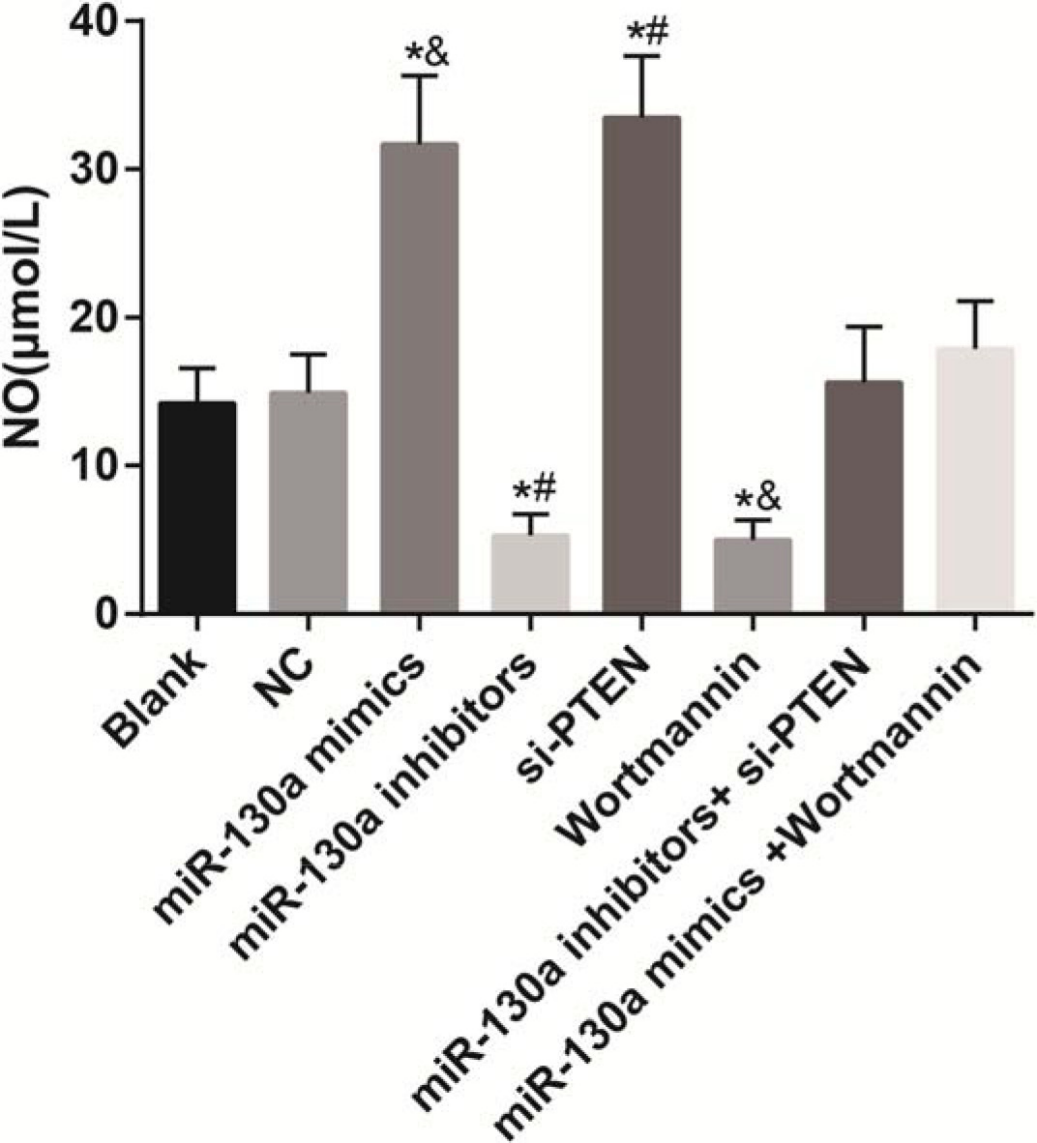
The release of NO in HCAECs among eight groups Note: HCAECs, human coronary artery endothelial cells; NO, nitric oxide; *P < 0.05 compared with the blank and the NC groups; ^#^*P* < 0.05 compared with the miR-130a inhibitors + si-PTEN group; ^&^*P* < 0.05 compared with the miR-130a mimics + Wortmannin group.

A NO kit was used for testing the NO content in Figure 11. No significant difference was found in the concentration of NO among the blank, NC, miR-130a inhibitors + si-PTEN and miR-130a mimics +Wortmannin groups (all *P* > 0.05). Compared with the blank and the NC groups, the concentrations of NO were increased in the miR-130a mimics and the si-PTEN groups while were decreased in the miR-130a inhibitors and the Wortmannin groups (all *P* < 0.05). Compared with the miR-130a inhibitors + si-PTEN group, the concentration of NO was decreased in the miR-130a inhibitors group while was increased in the si-PTEN group (both *P* < 0.05). Compared with the miR-130a mimics +Wortmannin group, the concentration of NO was increased in the miR-130a mimics group while was decreased in the Wortmannin group (both *P* < 0.05).

### Comparisons of serum cytokine levels in HCAECs among eight groups

There was no significant difference in the concentrations of IL-6, ICAM-1, IFN-γ, IL-1β, TNF-α, IL-12 and IL-17 between the blank and the NC groups (all *P* > 0.05). Compared with the blank and the NC groups, the concentrations of IL-6, ICAM-1, IFN-γ, IL- 1β, TNF-α, IL-12 and IL-17 were decreased in the miR-130a mimics and the si-PTEN groups while were increased in the miR-130a inhibitors and the Wortmannin groups (all *P* < 0.05). No significant difference was found in the concentrations of these inflammatory factors between the miR-130a inhibitors + si-PTEN and the miR-130a mimics +Wortmannin groups (all *P* > 0.05). The concentrations of these inflammatory factors in the miR-130a inhibitors group were higher than those in the miR-130a inhibitors + si-PTEN group, and in the si-PTEN group were lower than those in the miR-130a inhibitors + si-PTEN group (all *P* < 0.05). The concentrations of these inflammatory factors in the miR-130a mimics group were lower than those in the miR-130a mimics + Wortmannin group, and in the Wortmannin group were higher than those in the miR-130a mimics + Wortmannin group (all *P* < 0.05) (Table 4).

**Table 4:** The concentrations of cytokines in each group

Group	IL-6(ng/ml)	ICAM-1(ng/ml)	IFN-γ(pg/ml)	IL-1β(pg/ml)	TNF-α(pg/ml)	IL-12(pg/ml)	IL-17(pg/ml)
Blank	57.88 ± 7.66	21.33 ± 3.64	70.15 ± 5.01	2.27 ± 0.59	11.56 ± 3.73	235.24 ± 10.52	312.76 ± 24.18
NC	56.06 ± 8.15	20.59 ± 3.26	70.43 ± 4.92	2.16 ± 0.61	12.35 ± 4.03	236.98 ± 11.25	330.09 ± 28.51
miR-130a mimics	32.59 ± 6.45*^&^	8.55 ± 1.67*^&^	49.66 ± 4.27*^&^	1.04 ± 0.49*^&^	4.86 ± 1.09*^&^	135.06 ± 15.70*^&^	217.32 ± 22.19*^&^
miR-130a inhibitors	102.15 ± 8.60*^#^	36.78 ± 6.55*^#^	134.72 ± 7.48*^#^	3.71 ± 0.89*^#^	24.05 ± 3.99*^#^	398.71 ± 30.14*^#^	566.38 ± 37.21*^#^
si-PTEN	29.94 ± 5.87*^#^	8.02 ± 1.59*^#^	46.23 ± 4.60*^#^	0.96 ± 0.45*^#^	4.09 ± 0.92*^#^	129.57 ± 16.18*^#^	211.66 ± 21.27*^#^
Wortmannin	109.64 ± 8.89*^&^	40.25 ± 6.24*^&^	145.80 ± 7.19*^&^	3.76 ± 0.95*^&^	26.73 ± 3.87*^&^	418.66 ± 28.37*^&^	582.49 ± 34.52*^&^
miR-130a inhibitors+ si-PTEN	54.20 ± 7.91	21.91 ± 3.72	72.08 ± 4.67	2.11 ± 0.58	10.69 ± 3.45	227.47 ± 11.60	328.12 ± 25.68
miR-130a mimics + Wortmannin	66.84 ± 6.79	26.82 ± 4.11	73.98 ± 5.45	2.42 ± 0.68	10.13 ± 1.70	241.55 ± 20.18	335.60 ± 29.76

Notes: HCY, homocysteine; IL-6, interleukin-6; ICAM-1, Intercellular Adhesion Molecule 1; IFN-γ, interferon-γ; IL-1β, Interleukin-1β; TNF-α, interferon-α; IL-12, interleukin-12; IL-17, interleukin-17; HCAECs: human coronary artery endothelial cells;

* compared with the blank and the NC groups, *P* < 0.05,

# compared with the miR-130a inhibitors+ si-PTEN group, *P* < 0.05;

& compared with the miR-130a mimics + Wortmannin, *P* < 0.05.

## DISCUSSION

In the present study, we focused on the mechanisms of miR-130a regulating PI3K/Akt/eNOS signaling pathway in HCAECs injury and inflammatory responses. Our study demonstrated that miR-130a activates PI3K/Akt/eNOS signaling pathway to reduce the HCAECs injury and inflammatory responses by down-regulating PTEN.

In our study, we successfully used HCY to induce HCAEC injury and HCAEC-mediated inflammatory responses. HCY, an asulphur-containing endogenous amino acid, is produced in the methylation cycle of protein metabolism and participated in maintaining the cells redox balance [21]. HCY can directly or indirectly lead to vascular endothelial cell injury, promote the proliferation of vascular smooth muscle cells, affect the oxidation of low density lipoprotein, enhance platelet function, and promote the formation of thrombosis [22]. A previous study has also indicated that HCY has been recognized as an independent risk factor for CHD, which can induce injuries and an inflammatory responses in cardiac endothelial cells [23]. We found that the NO concentrations were lower than those in the control group after treatment with different doses of HCY, and with increasing HCY concentrations, the IL-6 and ICAM- 1 content in HCAECs increased gradually. HCY can generate reactive oxygen and a large number of radicals under oxidative stress, which can directly damage the endothelium, and the product can not only promote the degradation of NO but also inhibit the production and activity of nitric oxide synthase, which can cause the decreased synthesis of NO [24]. A study conducted by Dai et al. also revealed that HCY-induced reactive oxygen species up-regulate the expression and translocation of redox factor 1 via NADPH oxidase, and then redox factor 1 increases nuclear factor-kappa B activity, monocyte chemoattractant protein-1 secretion by human monocytes/macrophages and the production of multiple inflammatory cytokines [25].

Subsequently, we conducted an experiment to investigate the effects of miR-130a on the PI3K/Akt/eNOS signaling pathway and concluded that the PI3K/Akt/eNOS signaling pathway was inhibited in HCAECs following HCY treatment after transfection with the miR-130a inhibitor. Previous evidence has shown that miR-130a suppresses PTEN expression, leading to the activation of PI3K/Akt signaling [26], which was consistent with our results. Finally, we found that miR-130a may activate PI3K/Akt/eNOS signaling pathway following HCY treatment, and then successfully induce HCAEC injury and HCAEC-mediated inflammatory responses. MiR-130a has recently emerged as a key miR that inhibits cancer cell proliferation, invasion and migration by targeting other cellular proteins that promote cell proliferation or have oncogenic potential [27]. PI3K, known as a heterodimeric enzyme, plays an important role in proliferation and apoptosis, while Akt, a downstream serine-threonine kinase, transmits survival signals from growth factors. It was reported that Akt can activate eNOS, which can cause the production of NO [28]. NO, an important messenger molecule, is closely related to the inflammatory responses, oxidative stress and cell proliferation [29]. It has been found that the PI3K/Akt/eNOS signaling pathway is involved in cell growth, proliferation, differentiation, movement, energy storage and metabolism [28]. Moreover, PI3K activation leads to the phosphorylation of AKT, which can activate its downstream target proteins, such as Bad, Caspase9, nuclear factor-kappa B and Bax, by phosphorylation, and then regulates the proliferation, differentiation, apoptosis and migration of ECs [30]. Previous evidence has also demonstrated that activated nuclear factor-kappa B can enter into the nucleus and combine with the target genes related to inflammation and the immune response, leading to the release of a large number of inflammatory factors, such as IL-6 and ICAM-1 [31, 32]. Angiopoietin-1 (Ang1), a strong activator of intracellular PI 3′-kinase/Akt, was also demonstrated that it may be useful as an inhibitor of TNF-α-induced inflammation and cancer progression [9]. PTEN is deemed as a tumor suppressor gene that negatively regulates PI3K/Akt signaling [33]. The suppression of PTEN has been proved to reduce myocardial ischemic injury through activation of PI3K/Akt signaling [34]. Previous study has claimed that miR-130a could down-regulate the expression of PTEN in ECs [35]. Moreover, a study conducted by Wu and colleagues clarified that vascular endothelial dysfunction was associated with the inhibition of the PI3K/Akt/eNOS signaling pathway [36]. Thus, we suspected that miR-130a may inhibit the PI3K/Akt/eNOS signaling pathway to cause HCAEC injury and HCAEC-mediated inflammatory responses.

In conclusion, we provide compelling evidence that miR-130a could alleviate HCAECs injury and inflammatory responses by down-regulating *PTEN* and activating PI3K/Akt/eNOS signaling pathway, which can provide a new target for the treatment of CHD at the gene level. However, due to the limitations of our funds and time of our study, mature miR-130a, pre-miR-130a and pri-miR-130a used for determining the functions of HCY on up-regulating miR-130a were not conducted. Thus, the underlying mechanism requires further investigation.

## MATERIALS AND METHODS

### Cell culture

Culture bottles containing human coronary artery endothelial cells (HCAECs, purchased from ScienCell Research Laboratories, Carlsbad, CA, USA) were wiped and disinfected with 70% alcohol and then cultured in an incubator for 2 h. After the cells adhered to the wall, the original culture medium was replaced by fresh culture medium for continuing culture, and the cell morphology was observed under a microscope. The morphology of the cultured cells was consistent with the characteristics of endothelial cells. When the cells grown over 90% of the culture bottle, the cells were subcultured and frozen for further experiments. All the cells were divided into two groups, the case and control groups. Phosphate buffered saline (PBS) (1 mL) was added to the control group, while different doses of HCY (0.1, 0.25, 0.5 and l mmol/L) were added to the case group at room temperature for 20 min (6 wells in each group). When the cells largely adhered to the wall (approximately 2–3 h), the corresponding treatment was continued for 24 hours in culture. Culture medium and culture bottle were purchased from Hyclone Laboratories (Logan, UT, USA).

### Cell transfection

HCAECs treated with HCY (1 mmol/L) were inoculated in a 50 ml culture bottle, and the cells grew to 30–50% density in complete culture medium. Lipofectamine 2000 and microRNA-130a inhibitor were prepared in sterile Eppendorf tubes. The Lipofectamine 2000 solution was prepared with 1 μl Lipofectamine 2000 + 50 μl serum-free medium and placed at room temperature for 5 min, and the miR-130a inhibitor was prepared with miR-130a inhibitors (20 pM) + 50 μl serum-free medium and placed at room temperature for 20 min. Diluted miR-130a inhibitor was used to form a complex with liposomes (total volume: 100 μl). Next, the complex was evenly mixed and added into the 50 ml culture bottle containing cells for transfection. Then, the mixture was placed in a 37°C, 5% CO_2_ incubator. After culture for 6–8 h, the culture solution was changed to fresh complete culture medium for further culture for 24–48 h to gather the cells and extract the total protein. HCAECs in the logarithmic growth phase were divided into eight groups: the blank group (without any treatment), the negative control (NC) group (transfected with empty vector), the miR-130a mimics group (transfected with miR-130a analogue), the miR-130a inhibitors group (transfected with miR-130a inhibitors), the si-PTEN group (transfected with si-PTEN), the Wortmannin group (Wortmannin [a PI3K inhibitor] was incubated with the cells for 24 h), the miR-130a inhibitors + si-PTEN group (co-transfected with miR-130a inhibitors and si-PTEN) and the miR-130a mimics + Wortmannin group (after transfection with miR-130a analogue for 6–8 h, the culture solution was changed to complete culture medium, and then Wortmannin was also incubated with the cells for 24 h).

### Luciferase reporter gene assay

DNA extraction was conducted based on operating specification of TIANamp Genomic DNA Kit (Tiangen Biotech, Beijing, China). After that, the luciferase reporter vector was constructed, and luciferase assay kit (Bright-Glo™ Luciferase Assay System) of Promega Corporation (Madison, Wisconsin, USA) was applied for testing the activity of luciferase. After the 293T cell (Human renal epithelial cell line) was transfected for 48 h, the old culture medium was discarded, followed by PBS washing for two times. After washing, each well was added with 100 μL Passive Lysis Buffer (PLB), followed by slightly shaken at room temperature for 15 min, and the cell lysis solution was gathered. The procedure was set to preread for 2 s and read for 10 s with per injection volume of LARIIStop&Glo^®^ Reagent was 100 μL. The prepared LAR II, Stop &Glo^®^ reagent and the luminotron or lighting slab added into cell lysis solution were placed into biological luminescence detector. The procedure run and the data were saved after the end of the fluorescent reading.

### Real-time quantitative polymerase chain reaction (RT-qPCR)

The cell culture medium from a 6-well plate was placed into six centrifuge tubes and centrifuged at 1200 rpm for 10 min. The supernatant (500 ml) was used to extract RNA, and the total RNA was extracted using a miRNeasy Mini Kit (Qiagen, Dusseldorf, Germany). The RNA samples (5 μl) were diluted 20 times with ultrapure RNase-free water, and the absorption value was read at 260 nm and 280 nm on an ultraviolet spectrophotometer to test the concentration and purity of the RNA. An OD260/OD280 ratio between 1.7~2.1 indicated sufficient purity for follow-up experiments. The cDNA template was synthesized by reverse transcription using a PCR amplification instrument, and RT-qPCR was conducted with an ABI7500 quantitative PCR instrument. The reaction conditions were: pre-denaturing (95°C for 10 min) and then 40 cycles of denaturing (95°C for 10 s), annealing (60°C for 20 s), and elongation (72°C for 34 s). The primers used in the reaction are shown in Table 5. U6 was regarded as an internal reference of miR-130a, and β-actin was regarded as an internal reference of PTEN, PI3K, AKT and eNOS mRNA expressions. The 2^−ΔΔCt^ method was used to represent the ratios of the target genes between the test group and the control group, and the formula was ΔΔCT = ΔCt _test group_ - ΔCt _control group_ and ΔCt = Ct _target gene_ - Ct _inference gene_. Ct was the number of amplification cycles that had occurred when the real-time fluorescence intensity of the reaction reached the set threshold. The experiment was conducted in triplicate.

**Table 5:** Primer sequences of genes

Gene	Forward primer	Reverse primer
microRNA-130a	5′-CAGTGCAATGTTAAAAGGGCAT-3′	5′-TAGAGTGAGTGTAGCGAGCA-3′
U6	5′-GCTTCGGCAGCACATATACTAAAAT-3′	5′-CGCTTCACGAATTTGCGTGTCAT-3′
PTEN	TGGAAAGGGACGAACTGGTG	CATAGCGCCTCTGACTGGGA
PI3K	CCACGACCATCATCAGGTGAA	CCTCACGGAGGCATTCTAAAGT
AKT	ACGATGAATGAGGT GTCTGT	TCTGCTACGGTGAAGTTGTT
eNOS	TGACCCTCACCGATACAACA	TCTGGCCTTCTGCTCATTTT
β-actin	5′-CCTGTACGCCAACACAGTGC-3′	5′-ATACTCCTGCTTGCTGATCC-3′

Notes: PTEN, Phosphatase and tensin homolog; PI3K, Phosphatidylinositol 3-kinase; Akt, Protein kinase B; eNOS, endothelial nitric oxide synthase.

### Western blotting

After removing the cultured cells, the cells were washed with pre-cooled PBS buffer for 3 times. After washing, lysis buffer (RIPA Buffer of Pirece company) was added (100 μl/50 ml) to lyse the cells, and then the cells were placed on ice for 30 min, followed by a 12000 rpm centrifugation at 4°C for 10 min. Then, the supernatants were stored separately in 0.5 ml centrifuge tubes at −20°C. Bovine serum albumin (BSA) (2 μg/μl) was diluted in PBS successively at concentrations of 20 μg/μl, 15 μg/μl, 10 μg/μl, 5 μg/μl, 2.5 μg/μl and 0 μg/μl. After the amount of BCA detection reagent needed was calculated according to the number of samples, reagents A and B from the BCA kit (Pierce) were prepared at a 50:1 ratio. The lysates (2 μl) were diluted in double-distilled water (18 μl) (each sample in 2 wells). In a 96-well plate, 200 μl of detection solution were added to each well, and the diluted standard and samples to be tested were added to the wells (10μl/well), followed by light shaking. Then, the sample was incubated at 37°C for 30 min, cooled and placed at room temperature. The OD values of the samples were detected at a wavelength of 490, and the standard curve was drawn to calculate the concentrations of the proteins in each sample, and then the sample was stored at −70°C. The samples were first electrophoresed at 60 v, and then the voltage was changed to 120 v when the samples reached the separation gel, followed by electrophoresis (conducted at 4°C) for 1–2 h. After electrophoresis, the proteins weretransferred onto polyvinylidene fluoride (PVDF) membranes for 2 h (conducted at 4°C). The PVDF was removed from the transfer apparatus, and the samples were blocked with 5% evaporated milk in tris-buffered saline-tween (TBST) and incubated at room temperature for 1–2 h. Primary antibodies against PTEN (1:1000, A2B1, Santa Cruz Biotechnology, California), PI3K (1:1000, ab86714, abcam, USA), p-PI3K (1: 1,000, cs-4228, Cell Signaling, USA), Akt (1:1,000, cs- 9272, Cell Signaling, USA), p-Akt (1:1,000, cs-9271, Cell Signaling, USA), eNOS (1:1000, ab76198, abcam, USA) and p-eNOS (1:500, ab76199, abcam, USA) were incubated with the samples at 4°C overnight, followed by three TBST washes (each time for 10 min). After washing, the corresponding secondary antibody (sheep anti rabbit secondary antibody labeled by HRP [l:5000, A0208, Beyotime, Shanghai, China]) was added and the membrane was incubated at room temperature for 1 h, followed by three TBST washes (each time for 10 min). The band was visualized by chemiluminescence, X-ray exposure, developing, and photographic fixing to analyze the data.

### Methyl thiazolyl-tetrazolium (MTT) assay

The cells in the logarithmic growth phase were extracted and washed twice with PBS. Then, the cells were digested with trypsin and suspended in a single-cell suspension using a pipette. A cell counter was used in our study. The cells were inoculated in 96-well plates with 3×10^3^–6×10^3^ cells per well. The volume of added cells in each well was 200 μl, and six duplicate wells were used. The 96-well plate was placed in a 37°C, 5% CO_2_ incubator and cultured for 24–72 h with the addition of MTT solution (20 μl, 5 mg/ml, Sigma, St. Louis, USA) for developing. Then, after 4 h of incubation in a 37°C, 5% CO_2_ incubator, the cell culture was ended and the culture solution in the 96-well plate was discarded. Then, dimethyl sulfoxide (DMSO) (150 μl) was added to each well with light shaking for 10 min to promote the dissolution of crystals. The optical density (OD) values of each well were detected by enzyme-linked immunosorbent assay (ELISA) after 24 h, 48 h and 72 h. The MTT curve was drawn with the absorbance values as longitudinal coordinates and the intervals as horizontal ordinates. The experiment was conducted in triplicate.

### Hoechst 33258 staining

Sterile cover slips were placed in a 24-well plate, and the cells in the logarithmic growth phase were extracted and digested by 0.25% trypsin-0.02% ethylenediamine tetraacetic acid (EDTA), followed by centrifugation. After centrifugation, the cells were resuspended in complete culture medium and inoculated in 24-well plates at500 μl per well (6 × 10^5^/well), followed by culture in a 37°C, 5% CO_2_ incubator. After the cells adhered to the wall, the cover slips were taken out, washed three times with PBS, and fixed with 4% paraformaldehyde (Wuhan BOSTER Co. Ltd. [Wuhan, China]) for 15 min, followed by three PBS washes (each time for 5 min). Hoechst 33258 fluid (Sigma Company, USA) was used for staining at room temperature for 5 min, followed by distilled water washing and air drying. Under the fluorescence microscope, 5 high magnifications of each group were randomly selected. Pyknotic, enriched and lightened nuclei were deemed apoptotic nuclei. The number of apoptotic nuclei in 10 fields of view under the fluorescence microscope was counted based on the number of apoptotic nuclei appearing in every 200 nuclei.

### Detection the release of Nitric oxide (NO)

The cells in the logarithmic growth phase were extracted and digested with 0.25% trypsin-0.02% EDTA, followed by centrifugation. After centrifugation, the cells were suspended in complete culture medium and inoculated in 24-well plates at 500 μl/well (6 × 10^5^ cells per well), followed by culture in a 37°C, 5% CO_2_ incubator. After the cells adhered to the wall, the NO secreted into the supernatant of the cell culture medium in each group was tested according to the operating steps of the NO kit (Shanghai Jining biological science and Technology Co., Ltd.). The computational formula was: NO content (μmol/L) = [(OD of determination tube)/(OD of blank tube)] × standard concentration (100 μmol/L) × dilution factor of the sample before the test.

### ELISA assay

The cells in the logarithmic growth phase were extracted and digested with 0.25% trypsin-0.02% EDTA, followed by centrifugation. After centrifugation, the cells were resuspended in complete culture medium and inoculated in 6-well plates (1 × 10^4^/each well), followed by culture in a 37°C, 5% CO_2_ incubator. After the cells adhered to the wall, the cell supernatant was gathered and centrifuged at 4°C to removing sediment, and then the supernatants were stored at −20°C. The concentrations of ICAM-1, IL-6, IFN-γ, IL-1β, TNF-α, IL-12 and IL-17 in the cell supernatants were detected according to the kit instructions. The well-prepared cell supernatant to be tested was diluted to 500 times. After mixture, 100 mL diluent and well-prepared titers were added into 96-well plate coated with antibody, respectively. Each group has three parallel wells and the liquid was discarded after incubation for 2 h. After washing, 200 mL enzymes labeled antibody was added and incubated for 2 h, followed by discard of the liquid. The 200 mL chromogenic substrate was added in well for incubation at dark for 20 min, followed by the addition of 50 mL stop buffer. The optical density (OD) value was tested at wavelength of 450 nm. The standard curve was drawn and the concentrations of cytokines were calculated. ICAM-1 and IL-6 kits were brought from Shenzhen Juying biotechnology company (Shenzhen, China), and IFN-γ, IL-1β, TNF-α, IL-12 and IL-17 were brought from Invitrogen Corporation (Grand Island, New York, USA).

### Statistical analysis

All the data were processed using SPSS 18 statistical software (SPSS Inc, Chicago, IL, USA). Comparisons among multiple groups were using one-way analysis of variance (ANOVA), and comparison between two groups was using the Least Significant Difference (LSD) method. *P* < 0.05 provided evidence of significant differences.
